# Early predictors of functional outcome in poor-grade aneurysmal subarachnoid hemorrhage: a systematic review and meta-analysis

**DOI:** 10.1186/s12883-022-02734-x

**Published:** 2022-06-30

**Authors:** Jordi de Winkel, Tim Y. Cras, Ruben Dammers, Pieter-Jan van Doormaal, Mathieu van der Jagt, Diederik W. J. Dippel, Hester F. Lingsma, Bob Roozenbeek

**Affiliations:** 1 Department of Neurology, Erasmus MC University Medical Center Rotterdam, Rotterdam, The Netherlands; 2 Department of Public Health, Erasmus MC University Medical Center Rotterdam, Rotterdam, The Netherlands; 3 Department of Neurosurgery, Erasmus MC University Medical Center Rotterdam, Rotterdam, The Netherlands; 4 Department of Radiology and Nuclear Medicine, Erasmus MC University Medical Center Rotterdam, Rotterdam, The Netherlands; 5 Department of Intensive Care Adults, Erasmus MC University Medical Center Rotterdam, Rotterdam, The Netherlands

**Keywords:** Intracranial aneurysm, Outcome, Poor-grade, Stroke, Subarachnoid hemorrhage

## Abstract

**Background:**

Patients with poor-grade aneurysmal subarachnoid hemorrhage (aSAH) often receive delayed or no aneurysm treatment, although recent studies suggest that functional outcome following early aneurysm treatment has improved. We aimed to systematically review and meta-analyze early predictors of functional outcome in poor-grade aSAH patients.

**Methods:**

We included studies investigating the association of early predictors and functional outcome in adult patients with confirmed poor-grade aSAH, defined as World Federation of Neurological Surgeons (WFNS) grade or Hunt and Hess (H–H) grade IV-V. Studies had to use multivariable regression analysis to estimate independent predictor effects of favorable functional outcome measured with the Glasgow Outcome Scale or modified Rankin Scale. We calculated pooled adjusted odds ratios (aOR) and 95% confidence intervals (CI) with random effects models.

**Results:**

We included 27 studies with 3287 patients. The likelihood of favorable outcome increased with WFNS grade or H–H grade IV versus V (aOR 2.9, 95% CI 1.9–4.3), presence of clinical improvement before aneurysm treatment (aOR 3.3, 95% CI 2.0–5.3), and intact pupillary light reflex (aOR 2.9, 95% CI 1.6–5.1), and decreased with older age (aOR 0.7, 95% CI 0.5–1.0, per decade), increasing modified Fisher grade (aOR 0.4, 95% CI 0.3–0.5, per grade), and presence of intracerebral hematoma on admission imaging (aOR 0.4, 95% CI 0.2–0.8).

**Conclusions:**

We present a summary of early predictors of functional outcome in poor-grade aSAH patients that can help to discriminate between patients with favorable and with unfavorable prognosis and may aid in selecting patients for early aneurysm treatment.

**Supplementary Information:**

The online version contains supplementary material available at 10.1186/s12883-022-02734-x.

## Background

Aneurysmal subarachnoid hemorrhage (aSAH) is a severe type of stroke that is associated with high morbidity and mortality [1, 2]. The clinical severity of aSAH is classified with the World Federation of Neurological Surgeons grade (WFNS) or Hunt and Hess grade (H–H), with a higher clinical grade indicating poorer prognosis. Patients with WFNS grade IV-V or H–H grade IV-V account for 18–24% of the SAH population and are referred to as “poor-grade patients” [3].

In agreement with current guidelines [4, 5], the majority of aSAH patients are being treated within 24 h﻿ [6]. Aneurysm treatment in poor-grade patients is often delayed until signs of neurological recovery to avoid providing futile therapies to moribund patients or adding to a high proportion of patients ending up in vegetative or functionally dependent state. However, subjecting poor-grade patients to delayed aneurysm treatment may result in rebleeding and potential loss of life. Especially, because rebleeding occurs most often in the hours following the ictus [7].

There is evidence that outcome following poor-grade aSAH is better than historically assumed. A recent meta-analysis investigating poor-grade patients has indicated that 76% of poor-grade patients may survive and 47% may experience favorable functional outcome [3]. In addition, some studies reported that emergency aneurysm treatment reduced the risk of rebleeding [3, 8] and improved functional outcome [8]. Other studies did not find improved functional outcome with aneurysm treatment within 24 ﻿h [3, 9, 10].

In conclusion, there is a need to identify early predictors of functional outcome to improve patient selection for (early) aneurysm treatment to avoid unnecessary rebleeding. Many predictors of functional outcome have been identified, but these have not been confirmed in a poor-grade population. In this systematic review and meta-analysis, we aimed to investigate early predictors of functional outcome in poor-grade aSAH patients.

## Methods

We conducted a systematic review and meta-analysis according to the Preferred Reporting Items for Systematic reviews and Meta-Analyses (Additional file 1:  Table 1) [11]. The study protocol was registered with the International Prospective Register of Systematic Reviews prior to study eligibility selection and was published on 08/13/2020 (available via: https://www.crd.york.ac.uk/prospero/display_record.php?ID=CRD42020198603). We developed a comprehensive search strategy with the aid of a medical information specialist to systematically search Embase, Medline, Google Scholar, Web of Science Core Collection, and the Cochrane Central Register of Controlled Trials (Additional file 2: Methods 1). We searched from inception to present date and limited our search to peer-reviewed articles written in English. We conducted our primary search on 05/25/2020 and performed a re-run on 11/30/2020. Potentially eligible articles after title and abstract screening underwent full-text review (Additional file 3: Methods﻿ 2). We evaluated the bibliography of eligible studies for additional references. The selection process was recorded using Endnote X9 software.

We performed data extraction with a data extraction form (available upon request). We contacted the corresponding authors in case of missing data. We performed quality assessment with the Quality In Prognosis Studies (QUIPS) tool for quality assessment [12]. Risk of bias (ROB) plots were created with the robvis ROB visualization tool [13]. A detailed description of the criteria to reach the final verdict on ROB is given elsewhere (Additional file 4: Methods 3). We performed the process of study selection, data extraction and quality assessment blinded and independently (J.W.,T.Y.C.). Any disagreements were solved by consulting a third reviewer (B.R. or H.F.L.).

The primary outcome was favorable functional outcome measured with the Glasgow Outcome Scale (GOS) or the modified Rankin Scale (mRS). We did not define favorable outcome or the time of outcome measurement (i.e., some studies defined favorable outcome as a mRS of 0–2 at 6 months, while others defined favorable outcome as a mRS of 0–3 at 1 year).

We summarized study characteristics and reported them as means with standard deviations or medians with interquartile ranges. We performed a systematic review of early predictors of functional outcome. Furthermore, for predictors which were adequately reported and uniformly defined in multiple studies, we performed a meta-analysis and calculated pooled adjusted odds ratios (aOR) and 95% confidence intervals (CI) with random effects models. The results of the meta-analysis were described with Forest plots. When multiple studies reported results based on the same study population, we included the study with the largest sample size [14–20]. We accounted for heterogeneity in the study design by performing post-hoc subgroup analyses stratifying for length of follow-up, for studies with a favorable outcome definition mRS 0–2 or GOS 4–5, and for studies including patients who have received aneurysm treatment and studies including patients who have not received aneurysm treatment. We defined early follow-up as follow-up up to six months and late follow-up as beyond six months after SAH. We assessed between-study heterogeneity with Higgin’s & Thompson’s *I*^2^ and influence plots, and publication bias by analyzing funnel plots and Eggers’ regression test for funnel plot asymmetry. We adjusted for publication bias with the trim-and-fill method [21]. We did not assess publication bias in meta-analyses including less than five studies. To offer a complete overview of available prognostic research, any study that was not eligible for meta-analysis is summarized separately in a descriptive manner. We performed analysis with R software (version 3.6.3, *meta* package version 5.1.1, *metafor* package version 3.0.2).

## Results

We included 27 studies (*n* = 3287) that met our selection criteria in our review (Additional file 5: Fig. 1) [14–20, 22–41]. Year of publication ranged from 1996 to 2020 (Additional file 6: Table 2). We did not identify additional studies through bibliographical review.

The median duration of follow up was 6 months (IQR 3–12, Table 1), the median sample size of the multivariable analysis was 104 (IQR 80–154), and 76% of patients received aneurysm treatment. One study did not report on how many patients were provided aneurysm treatment and in one study aneurysm treatment was not provided at all. Most studies had a single center (*n* = 17, 63%) and retrospective (*n* = 24, 89%) design﻿.

**Table 1:** Summary of study characteristics

Study characteristic	
Patients in multivariable analysis – median (IQR)	104 (80–154)
Study design – n (%)	
Single-center	17 (63)
Multi-center	7 (26)
Retrospective	24 (89)
Prospective	3 (11)
Cohort	26 (96)
Case–control	1 (3)
Length of follow up – n (%)	
median (IQR)	6 (3–12)
< 6 months	15 (56)
> 6 months	11 (41)
Not reported	1 (3)
Definition of favorable outcome by mRS – n (%)	19 (70)
mRS 0–1	1 (4)
mRS 0–2	9 (33)
mRS 0–3	8 (30)
mRS 0–4	1 (4)
Definition of favorable outcome by GOS – n (%)	7 (26)
GOS 4–5	8 (30)
No definition of favorable outcome reported – n (%)	1 (4)
Studies that have exclusively included patients that were WFNS or H–H grade V – n (%)	5 (19)
Mean percentage of patients that received aneurysm treatment^a^ – (%)	76
Studies that included only patients that received aneurysm treatment^a^ – n (%)	16 (60)

*IQR* interquartile range, *mRS* modified Rankin Scale, *GOS* Glasgow Outcome Scale, *WFNS* World Federation of Neurological Surgeons, *H–H* Hunt and Hess

^a^One study did not report the number of patients that received aneurysm treatment

The studies investigated 82 early predictors of functional outcome with multivariable regression analysis. Taking into account predictor definition, reporting quality, and, if present, categorization we were able to conduct a systematic review of sixteen predictors and meta-analysis of nine predictors. We meta-analyzed age per decade increase, sex, clinical grade on admisssion, pupillary light reflex, clinical improvement before aneurysm treatment, modified Fisher grade, and presence of hydrocephalus, intraventricular hemorrhage (IVH), and intracerebral hematoma (ICH) on admission imaging (Additional file 7: Table 3). Aneurysm size, aneurysm location, Glasgow Coma Scale (GCS), Fisher grade, other concomitant bleeding, brain infarction on admission imaging, and leukocytosis were suitable for systematic review (Additional file 8: Table 4).

**Fig. 1:**
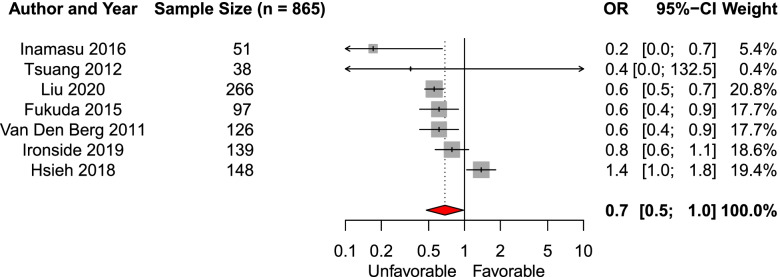
Forest plot of the effect of age (per decade increase) on functional outcome Abbreviations: *OR* Adjusted odds ratio, *CI* Confidence interval

We included fifteen studies in the systematic review of the early predictor age. Seven studies were eligible for meta-analysis (*n* = 865). The likelihood of favorable functional outcome decreased with older age (per decade, pooled aOR 0.7, 95% CI 0.5–1.0, Fig. 1) [24–27, 31, 38, 39]. We observed moderate funnel plot asymmetry, and after adjusting for publication bias the effect of age was no longer significant (*p* = 0.10, Additional file 9: Fig. 2A-B). In the eight studies not eligible for meta-analysis older age was often associated with worse functional outcome [14, 15, 19, 29, 35–37, 41].

**Fig. 2:**
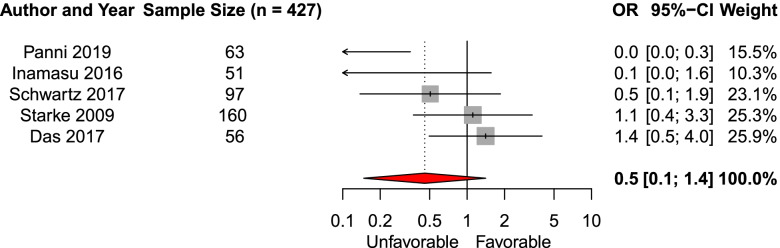
Forest plot of the effect of male sex on functional outcome Abbreviations: *OR* adjusted odds ratio, *CI* Confidence interval

We included six studies in the systematic review investigating the effect of sex on functional outcome. Five studies (*n* = 427) were eligible for meta-analysis [15, 23, 26, 32, 35]. We did not observe an association between sex and the likelihood of favorable functional outcome (pooled aOR 0.5, 95% CI 0.1–1.4, Fig. 2). One study was not eligible for meta-analysis and found no association between age and functional outcome [41].

**Fig. 3:**
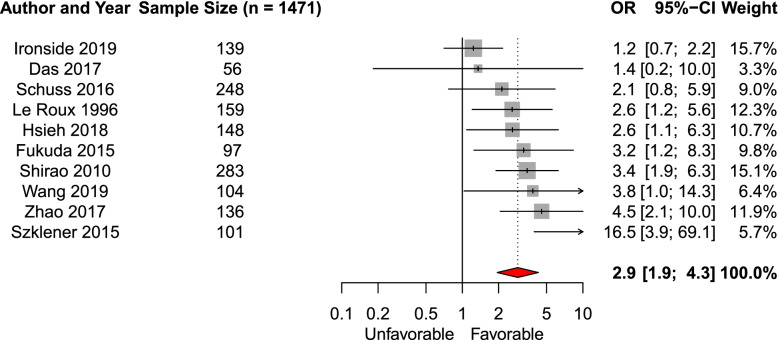
Forest plot of the effect of admission WFNS grade and H–H grade IV versus V on functional outcome Abbreviations: *OR* adjusted odds ratio, *CI* Confidence interval

We included thirteen studies in the systematic review of clinical grade on the likelihood of favorable functional outcome. Ten studies (*n* = 1471) were eligible for meta-analysis. The pooled aOR of WFNS grade IV versus V and H–H grade IV versus V was 2.9 (95% CI 1.9–4.3, Fig. 3) [17, 23–25, 27, 30, 34, 36, 37, 40]. The effect estimate for clinical grade was similar when including only studies investigating WFNS grade and not H–H grade [17, 24, 34, 36, 40]. In three studies not included in the meta-analysis higher clinical grade was associated with poorer outcome [14, 16, 18]﻿.

**Fig. 4:**
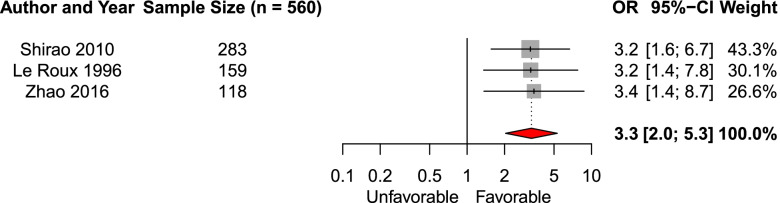
Forest plot of the effect of presence of clinical improvement before aneurysm treatment on functional outcome Abbreviations: *OR* adjusted odds ratio, *CI* confidence interval

We included three studies (*n* = 560, (11%)) investigating the effect of clinical improvement before aneurysm treatment on the likelihood of favorable functional outcome [17, 30, 36]. The pooled aOR was 3.3 (95% CI 2.0–5.3, Fig. 4). Further, we reviewed GCS on admission as an early predictor. Three studies included in the systematic review reported an increased likelihood of favorable functional outcome with increasing GCS [15, 20, 31, 41].

**Fig. 5:**
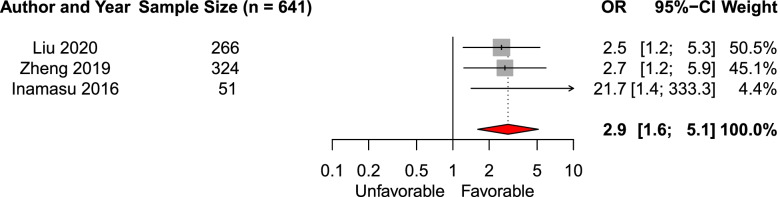
Forest plot of the effect of intact pupillary light reflex on admission on functional outcome Abbreviations: *OR* adjusted odds ratio, *CI* Confidence interval

We included three studies (*n* = 641) in the systematic review and meta-analysis of the effect of intact pupillary light reflex on admission [20, 26, 31]. The pooled aOR was 2.9 (95% CI 1.6–5.1, Fig. 5).

**Fig. 6:**
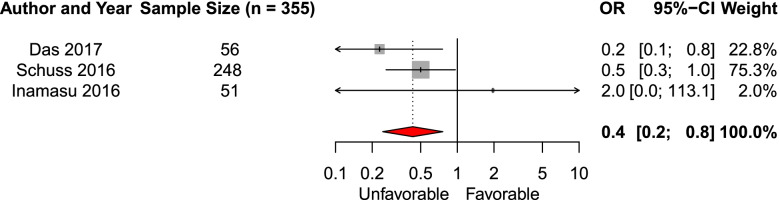
Forest plot of the effect of presence of intracerebral hematoma of functional outcome Abbreviations: *OR* adjusted odds ratio, *CI* Confidence interval

We included seven studies in the systematic review of the effect of presence of ICH on admission imaging on the likelihood of favorable functional outcome. Three studies (*n* = 355) were eligible for meta-analysis [23, 26, 34]. The pooled aOR was 0.4 (95% CI 0.2–0.8, Fig. 6). The remaining four studies did not report a significant effect of ICH on functional outcome [25, 28, 32, 41].

**Fig. 7:**
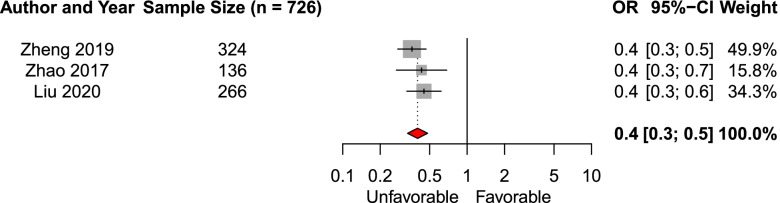
Forest plot of the effect of modified Fisher grade (per grade increase) on admission imaging on functional outcome Abbreviations: *OR* = adjusted odds ratio; CI = confidence interval

We included three studies (*n* = 726) in the meta-analysis of the effect of modified Fisher grade per grade on the likelihood of favorable functional outcome [18, 20, 31]. We found a pooled aOR of 0.4 (95% CI 0.3–0.5, Fig. 7). We included six studies in the systematic review investigating the effect of Fisher grade on functional outcome [19, 23, 25, 36, 37, 40, 41]. Three studies reported a significant association of higher Fisher grade with functional outcome.

**Fig. 8:**
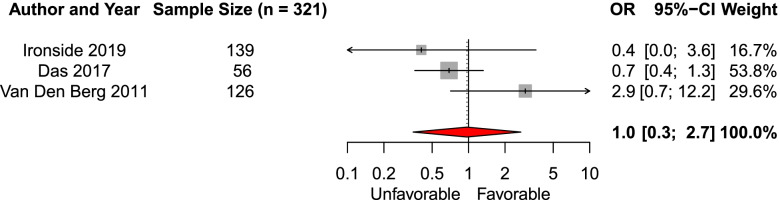
Forest plot of the effect of hydrocephalus on functional outcome Abbreviations: *OR* adjusted odds ratio, *CI* Confidence interval

We included five studies in the systematic review of the effect of presence of hydrocephalus before aneurysm treatment on functional outcome. Three studies (*n* = 321) were eligible for meta-analysis [23, 27, 39]. The pooled aOR was 1.0 (95% CI 0.3–2.7, Fig. 8). Two studies were not eligible for meta-analysis. Neither found a significant association with functional outcome [29, 40].

**Fig. 9:**
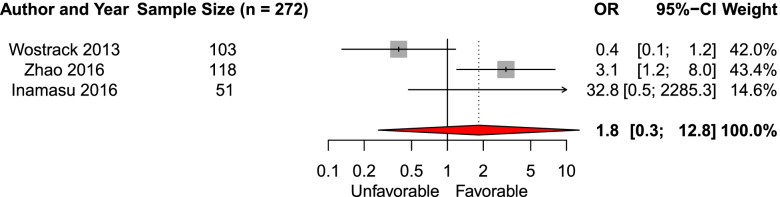
Forest plot of the effect of intraventricular hemorrhage on functional outcome Abbreviations: *OR* adjusted odds ratio, *CI* Confidence interval

We included seven studies in the systematic review of the effect of presence of IVH on admission imaging on the likelihood of favorable functional outcome. Three studies were eligible for meta-analysis (*n* = 272) [16, 26, 41]. The pooled aOR was 1.8 (95% CI 0.3–12.8, Fig. 9). Four studies were not eligible for meta-analysis and analyzed in with systematic review. Two found an association of IVH with functional outcome [30, 32, 40, 41].

Additionally, we conducted a systematic review of aneurysm size, aneurysm location, presence of brain infarction on admission imaging, leukocytosis, and other concomitant bleeding in relation to function outcome (Additional file 8: Table 4).

**Fig. 10:**
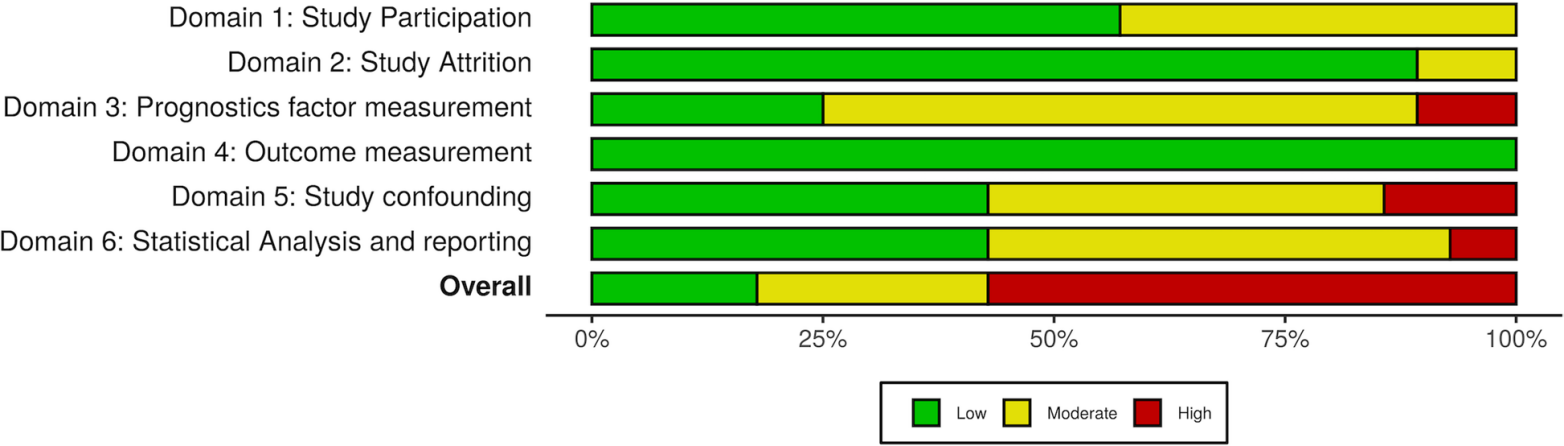
Risk of bias summary plot

We performed subgroup analyses for length of follow-up, for favorable outcome definition, and for studies including only patients that received aneurysm treatment for the predictors age, sex, and clinical grade, which showed similar results as the main analysis. The overall risk of bias as assessed with the QUIPS ROB tool for prognostic studies was high (Fig. 10, and Additional file 10: Fig. 3).

## Discussion

We systematically reviewed and meta-analyzed early predictors of functional outcome in poor-grade aSAH patients. In agreement with previous research, we confirmed that age, clinical grade, pupillary light reflex, presence of ICH, and modified Fisher grade were predictors of functional outcome [42–48]. In addition, we summarized available prognostic research of less well-known early predictors. In contrast to previous research, we did not find an association of functional outcome and sex, hydrocephalus, and IVH, and found little evidence of aneurysm size as an early predictor in this population [42–45, 47].

Overall, we found that higher level of consciousness and clinical improvement indicated better patient prognosis. Reassessing clinical condition after initial neurological resuscitation obtains more reliable prognostic estimates and may mandate re-evaluation of clinical management [49].

Not surprisingly, aSAH patients with intact pupillary light reflexes on admission had a greater likelihood of favorable functional outcome. However, seven of the included studies in the present systematic review excluded patients with absent brainstem reflexes [18, 24, 26, 30, 33, 38, 41]. A previous study advocated to add signs of brain stem herniation such as absent brainstem reflexes to the WFNS grade to improve prognosis prediction among grade V patients [50].

Many studies considered imaging-characteristics as predictors of functional outcome, with one-third of predictors evaluated being imaging-based. The widespread availability of imaging at baseline makes imaging-characteristics interesting for predicting prognosis. We observed that many studies used categorization and dichotomization, and applied different definitions for equal predictors. This has made the results of these studies unsuited for further meta-analysis. We advocate to adhere to the common data elements for SAH and unruptured intracranial aneurysms [51], and to limit categorization and dichotomization to enhance reproducibility and avoid losing valuable information within the data.

Nonetheless, we found that presence of ICH and modified Fisher grade were significantly associated with functional outcome. Presence of ICH was previously reported as a predictor of unfavorable outcome [52]. Prognosis of these patients may be intertwined with rapid hematoma evacuation. Denying surgical treatment because of poor expected outcome could lead to a self-fulfilling prophecy. Although, we found no association of other imaging-characteristics with functional outcome, due to lacking high-quality evidence, their prognostic value remains undetermined.

This study is strengthened by the comprehensive summary of prognostic research of early predictors of functional outcome in a poor-grade aSAH population. We confirmed predictors of outcome in a poor-grade population, and showed that there is an absence of high-quality prognostic evidence. Another strength is to limit study eligibility to those that performed multivariable analyses. This has added to the validity of the results.

Several limitations must be considered while interpreting this study. Methodological variation between the included studies led to considerable heterogeneity. For example, there was no uniform definition of favorable outcome in the included studies. This has made interpretation of the results of the meta-analysis more complicated and could have led to biased results.

Also, specific patient-characteristics may have guided the decision whether or not to pursue aggressive management. This may affect functional outcome and could have affected estimated predictor effects. However, most studies applied an aggressive treatment policy. This is illustrated by the high percentage of patients (76%) that received aneurysm treatment. Subgroup analysis for studies with a favorable outcome definition of mRS 0–2 and GOS 4–5, and for studies including exclusively patients who received aneurysm treatment did not indicate different findings than in the main analysis.

The results of our study could be affected by publication and reporting bias. When present, we aimed to adjust for publication bias. Not all studies reported non-significant aORs and CIs leading to a possible overestimation of the effect size estimates. Attempts to request the authors to provide this information were not successful.

Ultimately, the quality of the included studies determine the reliability of the results. Most studies had a small sample size and a high ROB. Because of this, the results have to be interpreted with caution.

Nevertheless, in this study, we obtained more valid and more precise estimates of predictors of functional outcome in a poor-grade aSAH population and summarized prognostic research for future prospective research. To date, no other intervention than aneurysm treatment can effectively minimize the risk of aneurysmal rebleeding. Poor-grade patients often receive delayed aneurysm treatment. Poor-grade patients that are most likely to achieve favorable outcome may be candidates for early aneurysm treatment. We argue that the early predictors of functional outcome that we present in this study could aid patient selection to avoid unnecessary rebleeding. Improving patient selection for early aneurysm treatment can both benefit patient outcome and ensure optimal allocation of limited health care resources.

Nonetheless, it should be noted that average improved functional outcome does not equal individual patient benefit. Individual treatment (strategy) effects can vary within the population. To provide absolute estimates of individual treatment benefit we have to model for heterogeneity of treatment effects which can only be performed using randomized data [53]. First, larger prospective observational research is needed to confirm these predictors of functional outcome in a poor-grade aSAH population. Next steps would be to implement these predictors of outcome in a prediction rule for clinical practice to provide estimates of expected benefit of early versus delayed aneurysm treatment in terms of functional outcome.

## Conclusions

We found that WFNS and H–H grade IV as opposed to V on admission, lower modified Fisher grade, the absence of intracerebral hematoma, intact pupillary light reflexes, and clinical improvement before aneurysm treatment were predictors of favorable functional outcome in poor-grade aSAH patients. These predictors can help discriminate between poor-grade aSAH patients with favorable and with unfavorable prognosis and may aid in selecting patients for early aneurysm treatment. The present study can serve as a stepping-stone for future decision modeling research focusing on selecting poor-grade aSAH patients for early aneurysm treatment.

## Supplementary Information

**Additional file 1: Table 1.** PRISMA checklist.

**Additional file 2: Methods 1.** Search strategy.

**Additional file 3: Methods 2.** Eligibility criteria.

**Additional file 4: Methods 3.** Risk of bias criteria for final verdict.

**Additional file 5: Figure 1.** PRISMA Flow chart.

**Additional file 6: Table 2. **Study characterization.

**Additional file 7: Table 3.** Meta-analysis.

**Additional file 8: Table 4.** Systematic review.

**Additional file 9: Figure 2A.** Funnel plots of meta-analysis of age before trim-and-fill. **Figure 2B.** Funnel plots of meta-analysis of age after trim-and-fill.

**Additional file 10: Figure 3.** Risk of bias traffic light plot.

## Data Availability

The dataset supporting the conclusions of this article is included within the article and its additional files.

## Abbreviations

aOR: Adjusted odds ratio
aSAH: Aneurysmal subarachnoid hemorrhage
CI: Confidence interval
GCS: Glasgow Coma Scale
GOS: Glasgow Outcome Scale
H–H: Hunt and Hess
ICH: Intracerebral hemorrhage
IVH: Intraventricular hemorrhage
IQR: Interquartile range
mRS: modified Rankin Scale
QUIPS: Quality In Prognosis Studies
ROB: Risk of bias
WFNS: World Federation of Neurological Surgeons
