# Supplementary material for: Early predictors of functional outcome in poor-grade aneurysmal subarachnoid hemorrhage: a systematic review and meta-analysis
Source: BMC Neurol. 2022 Jun 30;22:239. doi: 10.1186/s12883-022-02734-x (PMC9245240; doi:10.1186/s12883-022-02734-x)
Supplement: Supplementary file 2 — Additional file 2: Methods 1. Search strategy. [file 12883_2022_2734_MOESM2_ESM.docx]

**Additional file 2; Methods 1.** Search strategy

| **Database searched** | **Via** | **Years of coverage** | **Records** | **After deduplication** |
| --- | --- | --- | --- | --- |
| Embase | Embase.com | 1971 - Present | 2787 | 2717 |
| Medline ALL | Ovid | 1946 - Present | 1845 | 291 |
| Web of Science Core Collection | Web of Knowledge | 1975 - Present | 989 | 56 |
| Cochrane Central Register of Controlled Trials | Wiley | 1992 - Present | 212 | 51 |
| Other sources: Google Scholar† | | | 200 | 49 |
| Total | | | 6033 | 3164†† |

† In Google Scholar only the 200 most relevant articles, before deduplicating, were screened for eligibility.

†† The number of records of the updated search do not necessarily agree with the flowchart. There are several possible reasons for this: (1) an article is no longer within the 200 most relevant articles before deduplicating on Google Scholar, (2) the automatically generated thesaurus terms of an article are changed by specialized employees in the Embase database, (3) ahead of print articles previously found by searching Medline Ovid Epub which are now included in Embase and do not longer agree with the stricter search term. The results displayed in the flowchart are the sum of the articles screened after both searches.

**Search strategy performed on 05/25/2020, updated on 11/30/2020**

*Embase.com*

('subarachnoid hemorrhage'/de OR (((aneurysm*) NEAR/9 (subarachnoid-hemorrhage*))):ab,ti,kw) AND ('Glasgow outcome scale'/de OR 'modified rankin score'/de OR (Glasgow-outcome* OR modified-rankin):ab,ti,kw) NOT ('juvenile'/exp NOT 'adult'/exp) NOT ([Conference Abstract]/lim AND [1800-2017]/py)

*Medline (Ovid)*

(exp Subarachnoid Hemorrhage/ OR (((aneurysm*) ADJ9 (subarachnoid-hemorrhage*))).ab,ti,kf.) AND (Glasgow Outcome Scale/ OR (Glasgow-outcome* OR modified-rankin).ab,ti,kf.) NOT ((exp Child/ OR Adolescent/) NOT exp Adult/)

*Web of Science*

TS=(((((aneurysm*) NEAR/8 (subarachnoid-hemorrhage*)))) AND ( (Glasgow-outcome* OR modified-rankin))) AND DT=(Article OR Review)

*Cochrane Central*

((((aneurysm*) NEAR/9 (subarachnoid-hemorrhage*))):ab,ti,kw) AND ( (Glasgow-outcome* OR modified-rankin):ab,ti,kw)

*Google Scholar (Top 200 relevant records)*

“aneurysmal subarachnoid hemorrhage” “Glasgow outcome”|“modified rankin”
