# Supplementary material for: Early predictors of functional outcome in poor-grade aneurysmal subarachnoid hemorrhage: a systematic review and meta-analysis
Source: BMC Neurol. 2022 Jun 30;22:239. doi: 10.1186/s12883-022-02734-x (PMC9245240; doi:10.1186/s12883-022-02734-x)
Supplement: Supplementary file 3 — Additional file 3: Methods 2. Eligibility criteria. [file 12883_2022_2734_MOESM3_ESM.docx]

**Additional file 3; Methods 2.** Eligibility criteria

*Inclusion criteria*

1. Studies that included adults with a subarachnoid hemorrhage (SAH) confirmed with computed tomography (CT) or lumbar puncture;
2. With presence of a saccular intracranial aneurysm (IA) demonstrated by digital subtraction angiography (DSA) or computed tomography angiography (CTa), which is identified as the origin of SAH;
3. Presenting in poor neurological condition (defined as Hunt and Hess grade IV-V or World Federation of Neurological Surgeons grade IV-V);
4. Studies that measured functional outcome with Glasgow Outcome Scale (GOS) or modified Rankin Score (mRS);
5. Studies that performed a multivariable regression analysis of the association of predictors during early admission and functional outcome.

*Exclusion criteria*

1. Studies that report to include aSAH patients in a non-consecutive manner and;
2. Studies that, in case of including all-grade aSAH patients, do not report and analyze poor-grade patients separately.
