# Supplementary material for: Early predictors of functional outcome in poor-grade aneurysmal subarachnoid hemorrhage: a systematic review and meta-analysis
Source: BMC Neurol. 2022 Jun 30;22:239. doi: 10.1186/s12883-022-02734-x (PMC9245240; doi:10.1186/s12883-022-02734-x)
Supplement: Supplementary file 4 — Additional file 4: Methods 3. Risk of bias criteria for final verdict. [file 12883_2022_2734_MOESM4_ESM.docx]

**Additional file 4; Methods 3.** Risk of bias criteria for final verdict

We used the following criteria to reach a final verdict on overall risk of bias (ROB): if at least one of the six QUIPS domains was scored as high ROB, or three domains deemed as moderate ROB, the overall ROB of the study was scored as “high”. If up to two domains were scored as moderate ROB, the overall ROB of the study was scored as “moderate”. In case a study had only one domain or less scored as moderate ROB, the overall ROB was scored as “low”.
