# Supplementary material for: Early predictors of functional outcome in poor-grade aneurysmal subarachnoid hemorrhage: a systematic review and meta-analysis
Source: BMC Neurol. 2022 Jun 30;22:239. doi: 10.1186/s12883-022-02734-x (PMC9245240; doi:10.1186/s12883-022-02734-x)
Supplement: Supplementary file 5 — Additional file 5: Figure 1. PRISMA Flow chart. [file 12883_2022_2734_MOESM5_ESM.docx]

**Additional file 5; Figure 1.** PRISMA Flow chart

Records identified through database searching
(n = 5683)

Records after duplicates removed (n = 2971)

Additional records identified after deduplicated re-run of search (n = 228)

Records screened for title and abstract
(n = 3199)

Records excluded
(n = 2894)

Full-text articles assessed for eligibility
(n = 305)

Reasons for exclusion (n = 277):

- Did investigate specifically or report separately poor-grade SAH (n = 196)
- No full-text available (n = 17)
- Predictors not pre-interventional (n = 13)
- No multivariate analyses (n = 17)
- Included non-aneurysmal, non-saccular or suspected aSAH

(n = 12)

- Other publication type (n = 6)
- Not in English (n = 7)
- Other (n = 9)

Studies included for analyses
(n = 28)
