# Supplementary material for: Early predictors of functional outcome in poor-grade aneurysmal subarachnoid hemorrhage: a systematic review and meta-analysis
Source: BMC Neurol. 2022 Jun 30;22:239. doi: 10.1186/s12883-022-02734-x (PMC9245240; doi:10.1186/s12883-022-02734-x)
Supplement: Supplementary file 6 — Additional file 6: Table 2. Study characterization. [file 12883_2022_2734_MOESM6_ESM.docx]

**Additional file 6; Table 2.** Study characterization

| **Study** | | **Source of data** | | **Participants** | | | **Outcome** | | | |
| --- | --- | --- | --- | --- | --- | --- | --- | --- | --- | --- |
| **Author and year of publication** | **Sample size** | **Period of study** | **Study design** | **Inclusion criteria** | **Exclusion criteria** | **Definition of poor-grade** | **Length of follow-up** | **Definition of favorable outcome** | **Scale** | **Predictors in multivariable analyses** |
| Anqi  2019^14^ | 248 | 2012-2017 | Single-center retrospective case-control study | - Tibetans; - Poor-grade SAH | - Diagnosis not confirmed radiologically; - <18 years; - Not choosing clipping; - HBG <110; - Lost to follow up | - H-H IV-V on admission | 6 months | 0-2 | mRS | - High hemoglobin concentration with propensity score matching for: age, hypertension, diabetes, coronary artery disease, hypercholesterolemia, prior aSAH, current smoking, and current alcohol abuse |
| Das  2017^15^ | 85 | 2012-2015 | Single-center retrospective cohort study | - Poor-grade SAH | - Angiogram negative SAH; - Patients who died before Tx; - Patients who refused Tx; - Improvement to H-H <4 before Tx | - H-H IV-V | Median 11.6 months | 0-3 | mRS | - Age - Sex - Preoperative H-H grade - Fisher grade - ICH - Hydrocephalus - Admission leukocyt level |
| Fukuda  2015^16^ | 97 | 2008-2013 | Single-center retrospective cohort study | - Poor-grade aSAH | - Patients who died before Tx; - Irreparable brainstem injury; - Limits on therapeutic interventions; - Died before evaluation for symptomatic vasospasm and chronic hydrocephalus | - WFNS IV-V | 6 months | 0-3 | mRS | - Age - Initial cerebral edema - Intrasylvian hematoma - WFNS grade |
| Hsieh  2018^17^ | 148 | 2006-2017 | Single-center retrospective cohort study | - Poor-grade aSAH; - Confirmed aneurysm; - Tx with clipping or coiling | - <18 years; - CTa negative SAH; - Rebleeding before Tx; - Tx-related complications with impact on outcome; - Non-aneurysmal SAH | - H-H IV-V | 3 months | 0-2 | mRS | - Age - H-H grade - Fisher grade - Focal mass - Venous delay phenomenon |
| Inamasu  2016^18^ | 51 | 2006-2013 | Single-center retrospective cohort study | - Poor-grade with GCS 3; - Received immediate treatment | - >80 years; - Prolonged or unwitnessed cardiac arrest; - Bilaterally fixed and dilated pupils after resuscitation; - Intractable hypotension; - Radiographic evidence of irreparable brain injury; - Limits on therapeutic interventions; - Patients presenting with convulsive seizures | - H-H V and   GCS 3 | 90 days | 4-5 | GOS | - Age - Sex - Intact respiratory pattern - Isocoric pupils - Intact pupillary light reflex - Vertebral aneurysm - Presence of IVH - Presence of ICH - Presence of pulmary edema - Admission SBP |
| Ironside  2019^19^ | 139 | 2000-2015 | Single-center retrospective cohort study | - >18 years; - Confirmed aSAH; - Poor-grade; - Functional outcome data available | - Patients who died during initial hospitalization | - WFNS IV-V | Mean 6.3 months | 0-2 | mRS | - Age - H-H grade - Acute hydrocephalus - EVD placement - Lack of cerebrospinalfluid shunt placement |
| Kaneko  2019^20^ | 71 | 2013-2016 | Single-center retrospective cohort study | - Poor-grade aSAH | - Absent brainstem reflexes after resuscitation; - Cardiopulmonary arrest upon arrival; - aSAH confirmation with lumbar puncture or autopsy imaging | - WFNS IV-V | 6 months | 0-2 | mRS | - Age - Sex - Time from onset to aneurysm treatment - Group 1: obstructive hydrocephalus - Group 2: massive ICH with brain herniation - Group 3: CT findings other than group 1/2 |
| Konczalla  2018^21^ | 139 | 2005-2014 | Single-center retrospective cohort study | - Confirmed SAH; - Comatose on admission | - Improvement in H-H; - <24 hours after EVD insertion or after Tx | - H-H V | 6 months | 0-2 | mRS | - Age - Bilateral intact corneal reflexes - Pupils equally round and reactive to light - Early hydrocephalus |
| Le Roux  1996^22^ | 159 | 1983-1993 | Single-center retrospective cohort study | - Confirmed SAH; - Poor-grade | - Absent brainstem reflexes or function | - H-H IV-V on admission | 6 months | 4-5 | GOS | - H-H grade - Blood glucose - Fibrin degradation products - Severity of IVH on admission - Low density on admission CT - No clinical improvement |
| Liu  2020^23^ | 266 | 2010-2012; 2013-2016 | Multi-center prospective cohort study | - 18-75 years; - Poor-grade; - Confirmed aSAH | - No aggressive resuscitation; - Conservative aneurysm treatment | - WFNS IV-V | 12 months | 0-3 | mRS | - Age - Pupil reactivity - GCS score - modified Fisher grade |
| Mocco  2006^24^ | 98 | 1996-2002 | Single-center retrospective cohort study | - Confirmed aSAH; - Poor-grade | - NR | - H-H IV-V | 12 months | 0-3 | mRS | - Age - Hyperglycemia - Worst H-H grade - Aneurysm size |
| Panni  2019^25^ | 63 | 2014-2016 | Single-center retrospective cohort study | - Confirmed aSAH; - Poor-grade | - NR | - Worst pre-obliteration WFNS grade IV-V | 12,5 months | 0-2 | mRS | - Sex - WFNS grade - Loss of consciousness global - Loss of consciousness persistent - Global volume - Cisternal bleeding - ICH bleeding - IVH bleeding |
| Ridwan  2019^26^ | 80 | 2001-2010 | Single-center retrospective cohort study | - Poor-grade; - Cardiac arrest with CPR due to SAH | - Non-aneurysmal SAH; - Irreparable brainstem injury despite EVD placement; - Cardiac arrest or CPR for other reasons during admission | - H-H V | Discharge | 0-3 | mRS | - Aneurysm location |
| Schuss  2016^27^ | 248 | 2004-2014 | Single-center retrospective cohort study | - Confirmed aSAH; - Poor-grade | - Clinical condition not justifying further treatment | - WFNS IV-V | 6 months | 0-2 | mRS | - Age - WFNS grade - Aneurysm size - Space-occupying hematoma - Signs of cerebral hematoma |
| Schwartz  2017^28^ | 97 | 2005-2010 | Single-center retrospective cohort study | - Poor-grade aSAH; - Tx <72 hours after ictus | - NR | - Worst pre-obliteration grade - WFNS IV-V | Median 3.2 years | 0-2 | mRS | - Age - WFNS grade - Sex - Statin therapy prior to SAH - Magnesium therapy prior to SAH - Cerebral infarction |
| Shirao  2010^29^ | 283 | 2003 | Multi-center retrospective cohort | - Poor-grade; - Confirmed SAH | - Not sufficiently stable for aneurysm treatment; - Limits on therapeutic interventions; - Parent vessel trapping; - Proximal occlusion of cerebral artery; - Both GDC embolization and surgical clipping | - WFNS IV-V | Discharge | 4-5 | GOS | - Age - Fisher grade - WFNS grade - Improvement in WFNS grade - Low density area on CT |
| Starke  2009^30^ | 160 | 1996-2005 | Single-center retrospective cohort study | - Poor-grade; - GCS of 5-12 with H-H IV-V | - H-H III with GCS ≥10 | - H-H IV-V | 12 months | 0-3 | mRS | - Age - Sex - GCS |
| Szklener  2015^31^ | 101 | 2001-2010 | Single-center retrospective cohort study | - Spontaneous SAH; - Poor-grade - Tx = conservative; | - <18 years; - Admission criteria >24 hours; - Non-aneurysmal; - Tx with clipping or coiling; - Serious comorbidity | - WFNS IV-V | 30 days | 0-4 | mRS | - Age - WFNS grade - Fisher grade - Leukocytosis |
| Tsuang  2012^32^ | 38 | 2007-2009 | Multi-center retrospective cohort study | - Confirmed aSAH; - PCT <6hrs after ictus & <2 hours after admission; - Poor-grade | - WFNS I-III after resuscitation; - Irreparable brainstem or thalamic injury; - Systemic hypotension that might have caused brain hypoperfusion; - Angiogram-negative SAH; - Non-aneurysmal SAH; - Absent PCT data | - Worst WFNS grade during ictus and intervention of IV-V | 3, 6 or 12 months | 4-5 | GOS | - Age - Prolonged mean transit time at bilateral thalami |
| Van Den Berg  2011^33^ | 126 | 2000-2007 | Multi-center retrospective cohort study | - Confirmed SAH; - Poor-grade | - NR | - WFNS V | 6 months | 4-5 | GOS | - Age - Hydrocephalus |
| Wang  2019^34^ | 104 | 2010-2017 | Single-center retrospective cohort study | - Poor-grade; - Tx <3 days after onset | - WFNS I-III before Tx; - Tx conservatively; - Follow-up <6 months | - WFNS IV-V after resuscitation | 6 months | 0-2 | mRS | - WFNS grade - Fisher grade - Low density area on CT - Hydrocephalus |
| Wostrack  2013^35^ | 103 | 2006-2010. | Multi-center retrospective cohort study | - Confirmed aSAH - Poor-grade; - No improvement <48 hours after EVD placement - Early occlusion and resuscitation | - Irreparable brainstem damage or severe systemic disorder | - WFNS V on admission | Median 20 months  (3-57) | 4-5 | GOS | - Age - Sex - Initial GCS - Initial pupillary status - Fisher grade - ICH - ICH volume - aSDH - IVH - Midline shift - Aneurysm location - Aneurysm size |
| Zhao  2015^36^ | 118 | 2010-2012  2012-2014 | Multi-center retrospective cohort study | - 18-75 years; - Poor-grade without improvement after resuscitation; - Tx with clipping; | - Neurological deterioration to poor-grade after admission; - Tx with coiling - Tx >21 days after ictus; - Follow up <6 months; - Withdrawal of care in ER | - WFNS IV-V after resuscitation | Mean 12.5 months (±3.4) | 0-1 | mRS | - Age - Middle cerebral artery aneurysm location - WFNS grade after resuscitation |
| Zhao  2016^37^ | 118 | 2010-2012  2012-2014 | Multi-center retrospective cohort study | - 18-75 years; - Poor-grade without improvement after resuscitation | - Died after resuscitation; - Withdrawal of care in ER; - Neurological deterioration to poor-grade after admission; - Tx >21 days after ictus; - Follow up <6 months | - WFNS IV-V after resuscitation | 6 months | 0-3 | mRS | - Age - WFNS grade after resuscitation - Brain herniation - IVH - Non-middle cerebral artery aneurysm location |
| Zhao  2017^38^ | 136 | 2010-2012  2012-2014 | Multi-center retrospective cohort study | - 18-75 years; - Confirmed aSAH; - Poor-grade; - Informed consent | - Abstaining management; - Non-aneurysmal SAH; - Tx before admission; - Pregnancy or lactating; - Loss of breath; - Severe systemic disorder with <1yr life-expectancy | - WFNS IV-V after resuscitation | 12 months | 0-3 | mRS | - Age - WFNS grade - Modified Fisher grade - Aneurysm neck size |
| Zheng  2018^39^ | 104 | 2010-2013 | Multi-center prospective cohort study | - >60 years; - Confirmed aSAH; - Poor-grade | - Good-grade aSAH before Tx; - Non-aneurysmal SAH; - Tx in non-centered hospital; - ICH not attributable to IA; - Life-expectancy <1yr | - Pre-obliteration WFNS grade IV-V | 12 months | 4-5 | GOS | - Age - WFNS grade - Fisher grade - Cerebral hernia |
| Zheng  2019^41^ | 324 | 2010-2012 | Multi-center prospective cohort study | - Confirmed aSAH; - Poor-grade; - Informed consent; - Complete follow up and clinical information | - Good-grade aSAH before Tx; - Non-aneurysmal SAH; - Tx in non-centered hospital; - ICH not attributable to IA; - Life-expectancy <1yr; - Pregnant or lactating women. | - (Pre-) admission grade - WFNS IV-V | 12 months | 0-3 | mRS | - Age - Model I: Breathing status - Model II: Pupillary reactivity, GCS score, modified Fisher grade |

**Abbreviations:** aSAH = aneurysmal subarachnoid hemorrhage; CPR = cardiopulmonary resuscitation; ER = emergency room; EVD = external ventricular drain; EVT = endovascular therapy; GCS = Glasgow Coma Scale; GOS = Glasgow Outcome Scale; H-H = Hunt and Hess grade; HBG = high hemoglobine; IA = intracranial aneurysm; ICH = intracerebral hematoma; LOC = loss of consciousness; mRS = modified Rankin Scale; NR = not reported; PCT = perfusion computed tomography; SDH = subdural hemorrhage; Tx = aneurysm treatment (clipping, coiling, no aneurysm treatment); WFNS = World federation of Neurological Surgeons grade.
