# Supplementary material for: Early predictors of functional outcome in poor-grade aneurysmal subarachnoid hemorrhage: a systematic review and meta-analysis
Source: BMC Neurol. 2022 Jun 30;22:239. doi: 10.1186/s12883-022-02734-x (PMC9245240; doi:10.1186/s12883-022-02734-x)
Supplement: Supplementary file 7 — Additional file 7: Table 3. Meta-analysis. [file 12883_2022_2734_MOESM7_ESM.docx]

**Additional file 7; Table 3.** Meta-analysis

| **Predictors** | **Studies (n)** | **Sample size (n)** | **Study - aOR (95% CI)** | **Pooled aOR (95% CI)** | **Heterogeneity** | **Publication bias**† |
| --- | --- | --- | --- | --- | --- | --- |
| Demographics | | | | |  |  |
| Age (per decade increase)†† | 7 | 865 | Inamasu 2016 – 0.2 (0.0 – 0.7)  Tsuang 2012 – 0.4 (0.0 – 132.5)  Liu 2020 – 0.6 (0.5 – 0.7)  van den Berg 2011 – 0.6 (0.4 – 0.9)  Fukuda 2015 – 0.6 (0.4 – 0.9)  Ironside 2019 – 0.8 (0.6 – 1.1)  Hsieh – 1.4 (1.0 – 1.8) | 0.7 (0.5-1.0)* | I^2^ = 83%, *p*<0.0001 | Moderate funnel plot asymmetry; adjusted *p*-value was 0.10 |
| Sex (male) | 5 | 427 | Das 2017 – 1.4 (0.5 – 4.0)  Starke 2009 – 1.1 (0.4 – 3.3)  Schwartz 2017 – 0.5 (0.1 – 1.9)  Inamasu 2016 – 0.1 (0.0 – 1.6)  Panni 2019 – 0.0 (0.0 – 0.3) | 0.5 (0.1-1.4) | I^2^ = 65%, *p*=0.17 | Moderate funnel plot asymmetry; adjusting did not affect effect size estimate |
| Clinical condition on admission | | | | |  |  |
| Admission WFNS score and H-H score IV (vs. V) | 10 | 1471 | Szklener 2015 – 16.5 (3.9 – 69.1)  Zhao 2017 – 4.5 (2.1 – 10.0)  Wang 2019 – 3.8 (1.0 – 14.3)  Shirao 2010 – 3.4 (1.9 – 6.3)  Fukuda 2015 – 3.2 (8.3 – 1.2)  Le Roux 1996 – 2.6 (1.2 – 5.6)  Schuss 2016 – 2.1 (0.8 – 5.9)  Hsieh 2018 – 2.6 (1.1 – 6.3)  Das 2017 – 1.4 (0.2 – 10.0)  Ironside 2019 – 1.2 (0.7 – 2.2) | 2.9 (1.9-4.3)* | I^2^ = 47%, *p*<0.05 | Egger’s test *p*=0.25; no funnel plot asymmetry |
| Presence of clinical improvement before aneurysm treatment | 3 | 560 | Zhao 2016 – 3.4 (1.4 – 8.7)  Le Roux 1996 – 3.2 (1.4 – 7.8)  Shirao 2010 – 3.2 (1.6 – 6.7) | 3.3 (2.0-5.3)* | I^2^ = 0% | Not performed |
| Intact admission pupillary light reflex | 3 | 641 | Liu 2020 – 2.5 (1.2 – 5.3)  Zheng 2019 – 2.7 (1.2 – 5.9)  Inamasu 2016 21.7 (1.4 – 333.3) | 2.9 (1.6-5.1)* | I^2^ = 11%, *p*=0.33 | Not performed |
| Admission imaging characteristics | | | | |  |  |
| Presence of hydrocephalus before aneurysm treatment | 3 | 321 | Ironside 2019 – 0.4 (0.0 – 3.6)  Das 2017 – 0.7 (0.4 – 1.3)  van den Berg 2011 – 2.9 (0.7 – 12.2) | 1.0 (0.3-2.7) | I^2^ = 47%, *p*=0.15 | Not performed |
| Presence of admission intraventricular hemorrhage | 3 | 272 | Wostrack 2013 – 0.4 (0.1 – 1.2)  Zhao 2016 – 3.1 (1.2 – 8.0)  Inamasu 2016 – 32.8 (0.5 – 2285.3) | 1.8 (0.3-12.8) | I^2^ = 80%, *p*<0.01 | Not performed |
| Presence of admission intercerebral hematoma | 3 | 355 | Das 2017 – 0.2 (0.1 – 0.8)  Schuss 2016 – 0.5 (0.3 – 1.0)  Inamasu 2016 – 2.0 (0.0 – 113.1) | 0.4 (0.2-0.8)* | I^2^ = 0% | Not performed |
| Modified Fisher grade (per grade) | 3 | 726 | Zheng 2019 – 0.4 (0.3 – 0.5)  Zhao 2017 – 0.7 (0.3 – 0.7)  Liu 2020 – 0.4 (0.3 – 0.6) | 0.4 (0.3-0.5)* | I^2^ = 0% | Not performed |

**Abbreviations:** aOR = adjusted odds ratio; CI = confidence interval; GCS = Glasgow Coma Scale; H-H = Hunt & Hess grade; MCA = middle cerebral artery; WFNS = World Federation of Neurological Surgeons grade.

† Publication bias was only assessed if more than or equal to 5 studies were included in the meta-analysis.

†† We removed one study from the meta-analysis of age because we were not able to confirm if the reported aOR was based on a dichotomized or continuous analysis of age^26^

* Significant
