# Supplementary material for: Early predictors of functional outcome in poor-grade aneurysmal subarachnoid hemorrhage: a systematic review and meta-analysis
Source: BMC Neurol. 2022 Jun 30;22:239. doi: 10.1186/s12883-022-02734-x (PMC9245240; doi:10.1186/s12883-022-02734-x)
Supplement: Supplementary file 8 — Additional file 8: Table 4. Systematic review. [file 12883_2022_2734_MOESM8_ESM.docx]

**Additional file 8; Table 4**. Systematic review

| **Study** | **Categorization** | **Sample Size** | **aOR (95% CI) – aOR (*p*-value)** |
| --- | --- | --- | --- |
| Age† | | | |
| Das 2017 | <60 (≥60) | 85 | 1.4 (0.5-4.1) |
| Mocco 2006 | ≥65(<60) | 98 | 0.2 (0.1-0.6) †† |
| Schwartz 2017 | ≤53 (53>) | 97 | 0.2 (0.1-0.5) †† |
| Schuss 2016 | NR | 248 | 0.4 (0.2-0.9) †† |
| Shirao 2010 | ≤ 49 (ref)  50-64  ≥65 | 283 | NA  0.4 (0.2-0.9) ††  0.2 (0.1-0.4) †† |
| Starke 2009 | ≤70 (ref)  >70 | 160 | NA  0.1 (0.0-0.6) †† |
| Szklener 2015 | Per tertile | 101 | 0.2 (*p*=0.0053) †† |
| Wostrack 2013 | Per year | 103 | 1.0 (*p*=0.003) †† |
| Zheng 2018 | 60-70 (ref)  >70 | 104 | NA  1.6 (0.9-2.7) |
| Sex† | | | |
| Wostrack 2013 | Male sex | 103 | 1.4 (*p*=0.360) |
| Clinical grade | | | |
| Mocco 2006 | Worst pre-operative H-H V | 98 | NR (*p*<0.001) †† |
| Zhao 2015 | Post resuscitation WFNS IV | 118 | 6.3 (2.4-16.6) †† |
| Zhao 2016 | Post resuscitation WFNS V | 118 | 0.2 (0.1-0.5) †† |
| GCS | | | |
| Liu 2020 | GCS | 266 | 1.3 (1.1-1.5) †† |
| Starke 2009 | GCS 3-4  GCS 5-7  GCS 8-9  GCS 10-12 (ref) | 160 | 0.0 (0.0-0.2) ††  0.0 (0.0-0.2) ††  0.1 (0.0-0.7) ††  NA |
| Wostrack 2013 | GCS | 113 | 0.9 (*p*=0.675) |
| Zheng 2019 | GCS | 324 | 1.2 (1.1-1.4) †† |
| Hydrocephalus† | | | |
| Konczalla 2018 | Presence of early hydrocephalus (defined as within 24 hours) | 139 | NS |
| Wang 2019 | Presence of Hydrocephalus | 103 | NR (*p*=0.849) |
| IVH† | | | |
| Le Roux 1996 | Severity of IVH on admission CT | 159 | 0.5 (0.2-0.8) †† |
| Panni 2019 | Presence of IVH 11% (expressed of percentage of global volume) | 63 | NS |
| Wang 2019 | Presence of IVH | 104 | NR (*p*=0.254) |
| Wostrack 2013 | Presence of IVH | 103 | 2.5 (*p*=0.005) †† |
| ICH† | | | |
| Hsieh 2018 | Focal mass | 148 | 0.40 (0.16-1.04) |
| Panni 2019 | Presence of ICH bleeding 10% (expressed of percentage of global volume) | 63 | NS |
| Wang 2019 | Presence of ICH | 104 | NR (*p*=0.447) |
| Wostrack 2013 | Presence of ICH  Presence of ICH (cm^3^) | 103 | 0.7 (*p*=0.456)  1.0 (*p*=0.625) |
| Other concomitant bleeding | | | |
| Fukuda 2015 | Intrasylvian hematoma | 97 | 5.8 (1.4-24.3) †† |
| Panni 2019 | Cisternal bleeding 64% (expressed of percentage of global volume) | 63 | 0.1 (0.0-0.7) †† |
| Wostrack 2013 | Presence of acute subdural hematoma | 103 | 1.0 (*p*=0.954) |
| Fisher grade | | | |
| Das 2017 | Fisher grade1-2  Fisher grade 3-4 (ref) | 85 | 1.7 (0.4-6.4)  NA |
| Hsieh 2018 | Fisher grade 4 | 148 | 0.7 (0.3-1.8) |
| Shirao 2010 | Fisher grade 1-2 (ref)  Fisher grade 3  Fisher grade 4 | 283 | NA  1.0 (0.1-8.6)  0.5 (0.1-4.6) |
| Szklener 2015 | Fisher grade | 101 | 0.2 (*p*=0.0051) †† |
| Wang 2019 | Fisher grade 1-2 | 103 | 12.1 (2.1-69.7) †† |
| Zheng 2018 | Fisher grade 1-2  Fisher grade 3-4 (ref) | 104 | 3.1 (1.1-8.4) ††  NA |
| Aneurysm size | | | |
| Schuss 2016 | Continuously (mm) | 248 | 1.1 (1.0-1.2) |
| Wostrack 2013 | Continuously (mm) | 103 | 1.0 (*p*=0.09) |
| Location of the ruptured aneurysm | | | |
| Inamasu 2016 | Vertebral artery | 51 | 11.5 (0.5-260.2) * |
| Ridwan 2019 | ACA/Acomm  ICA/Pcomm | 80 | NR (*p*=0.007) †† |
| Wostrack 2013 | MCA  ACA  ICA  Posterior circulation | 103 | 0.6 (*p*=0.322)  1.0 (*p*=0.912)  1.4 (*p*=0.503)  0.7 (*p*=0.502) |
| Zhao 2016 | MCA | 118 | 0.4 (0.1-1.0) †† |
| Brain infarction on admission imaging | | | |
| Le Roux 1996 | Presence of brain infarction | 159 | 0.1 (0.0-0.5) †† |
| Schwartz 2017 | Presence of brain infarction | 97 | 0.3 (0.1-1.0) †† |
| Leukocytosis§ | | | |
| Das | Leukocytosis (WBC >15x10^9^/L) |  | 3.1 (1.1-8.5) |
| Szklener | Leukocytosis (WBC >15x10^9^/L) |  | 6.2 (2.6-14.8) |

**Abbreviations:** aOR = adjusted odds ratio; ACA = anterior cerebral artery; Acomm = anterior communicating artery; CI = confidence interval; GCS = Glasgow Coma Scale; H-H = Hunt & Hess grade; ICH = intercerebral hematoma; IVH = intraventricular hemorrhage; MCA = middle cerebral artery; NA = not applicable; NR = not reported; NS = not significant; PC = posterior cerebral artery; Pcomm = posterior communicating artery; ref = reference category; VA = vertebral artery; WFNS = World Federation of Neurological Surgeons grade, WBC = White blood cell count.

† Because of categorization, dichotomization, poor or incomplete reporting these studies were not eligible to include in the meta-analysis.

†† Significant

* In this study in 17% of patients the aneurysm location was not verified with imaging due to poor clinical condition.

§ Although technically feasible we did not perform further meta-analysis of the predictor leukocytosis because of large methodological variation and because there were only two studies available.
