# Supplementary material for: Early predictors of functional outcome in poor-grade aneurysmal subarachnoid hemorrhage: a systematic review and meta-analysis
Source: BMC Neurol. 2022 Jun 30;22:239. doi: 10.1186/s12883-022-02734-x (PMC9245240; doi:10.1186/s12883-022-02734-x)
Supplement: Supplementary file 9 — Additional file 9: Figure 2A. Funnel plots of meta-analysis of age before trim-and-fill. Figure 2B. Funnel plots of meta-analysis of age after trim-and-fill. [file 12883_2022_2734_MOESM9_ESM.docx]

**Additional file 9; Figure 2A.** Funnel plots of meta-analysis of age before trim-and-fill

**
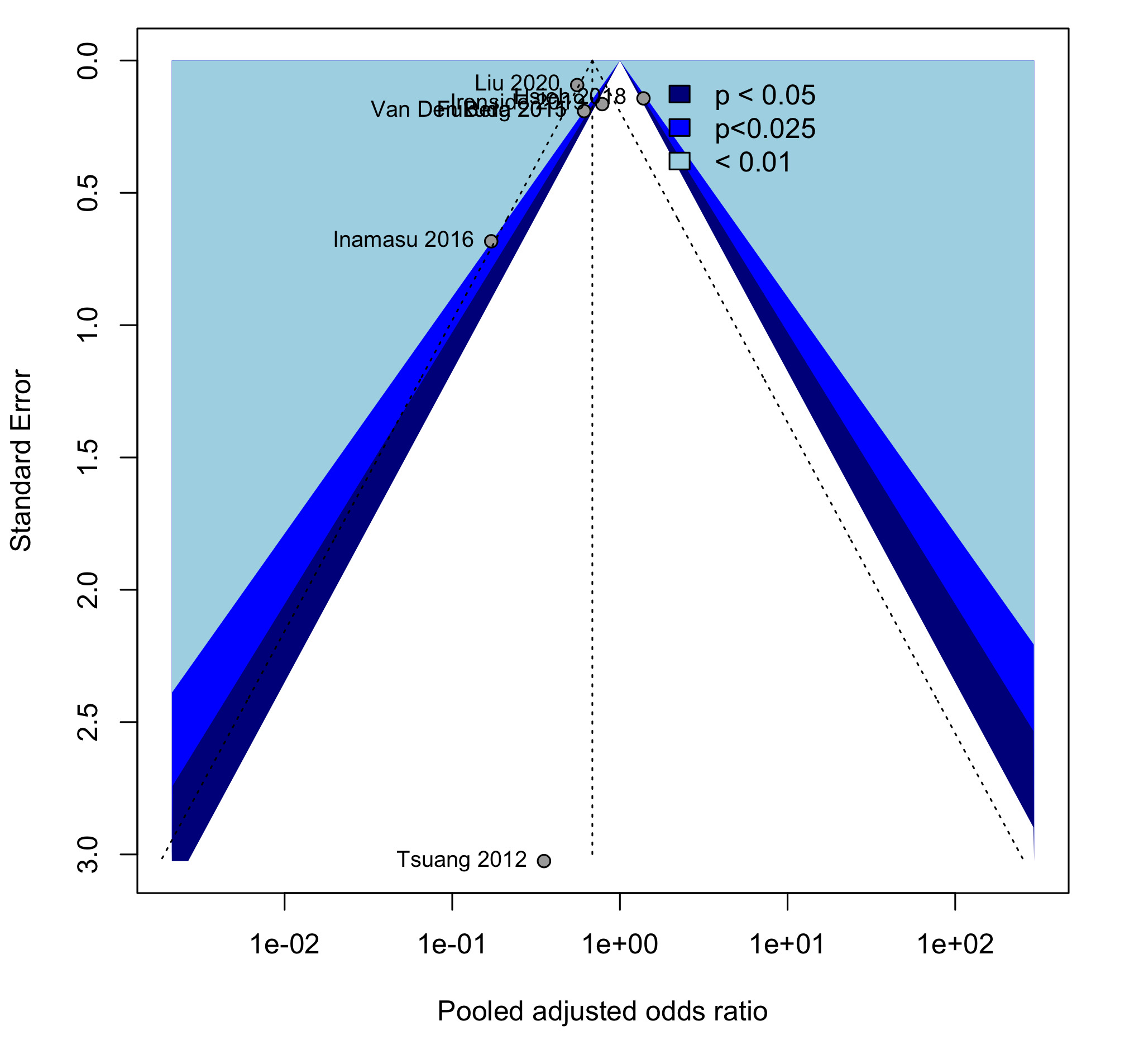
**

**Additional file 9; Figure 2B.** Funnel plots of meta-analysis of age after trim-and-fill

**
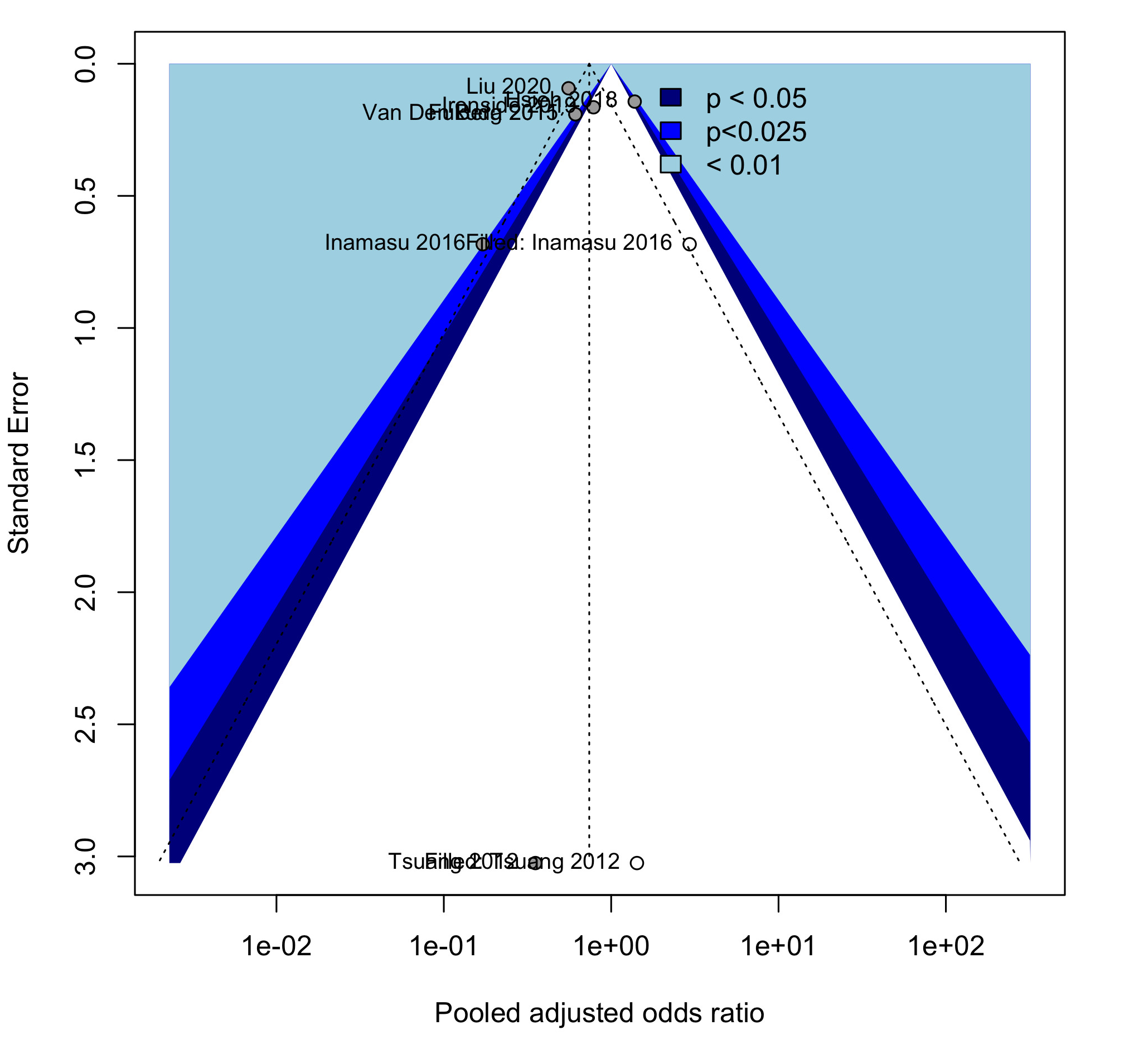
**
