# Supplementary figures and images for: Early predictors of functional outcome in poor-grade aneurysmal subarachnoid hemorrhage: a systematic review and meta-analysis
Source: BMC Neurol. 2022 Jun 30;22:239. doi: 10.1186/s12883-022-02734-x (PMC9245240; doi:10.1186/s12883-022-02734-x)

**Additional file 10; Figure 3.** Risk of bias traffic light plot


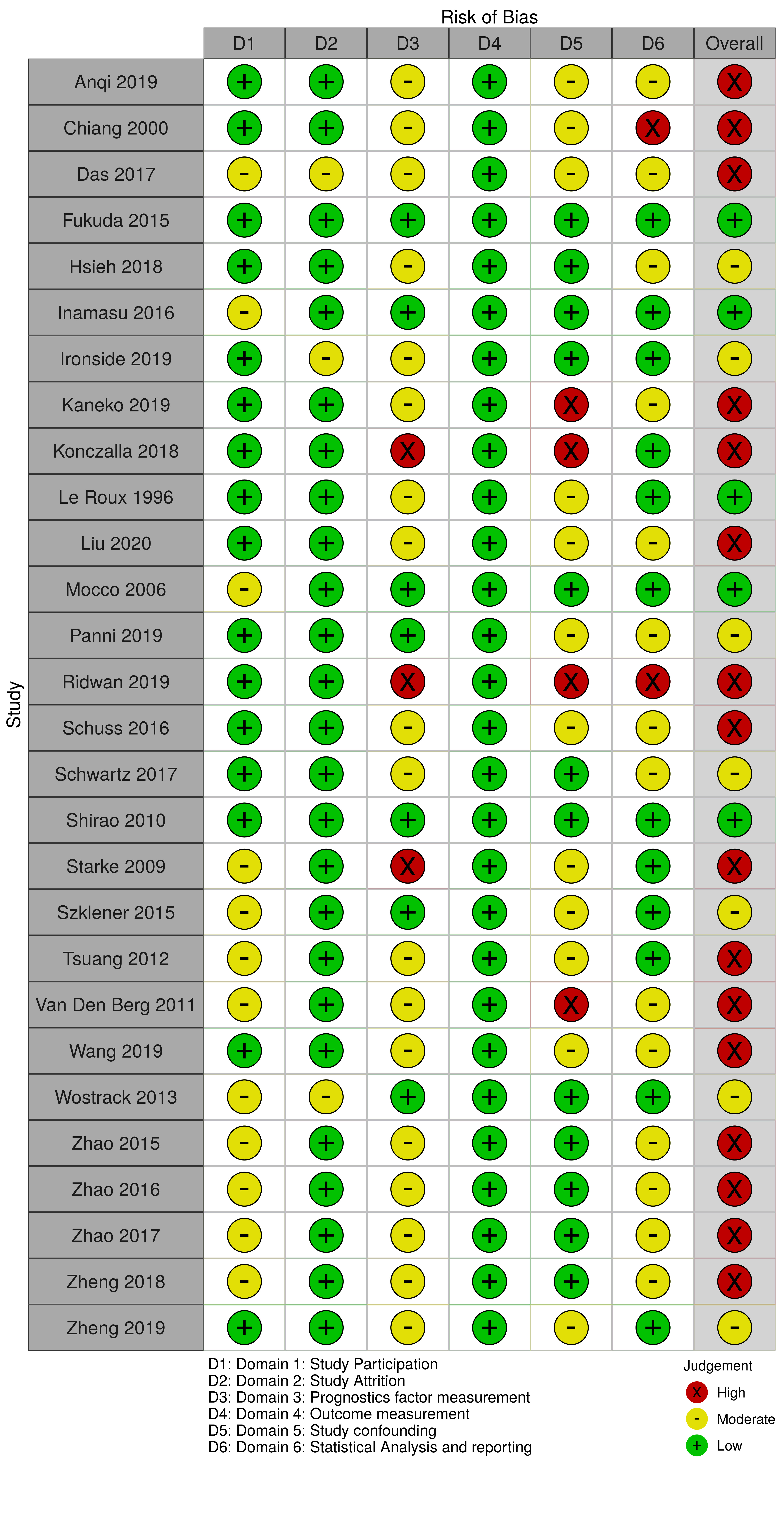

Supplement: Supplementary file 10 — Additional file 10: Figure 3. Risk of bias traffic light plot. [file 12883_2022_2734_MOESM10_ESM.docx]
